# Changes to Biceps and Supraspinatus Tendons in Response to a Progressive Maximal Treadmill-Based Propulsion Aerobic Fitness Test in Manual Wheelchair Users: A Quantitative Musculoskeletal Ultrasound Study

**DOI:** 10.1155/2021/6663575

**Published:** 2021-03-01

**Authors:** Mylène Leclerc, Cindy Gauthier, Rachel Brosseau, François Desmeules, Dany H. Gagnon

**Affiliations:** ^1^School of Rehabilitation, Faculty of Medicine, Université de Montréal, Québec, Canada; ^2^Pathokinesiology Laboratory, Center of Interdisciplinary Research in Rehabilitation of Greater Montreal, Institut universitaire sur la réadaptation en déficience physique de Montréal, Centre intégré universitaire de santé et de services sociaux du Centre-Sud-de-l'Ile-de-Montréal, QC, Canada; ^3^Orthopaedic Clinical Research Unit, Maisonneuve-Rosemont Hospital Research Center, QC, Canada

## Abstract

**Objective:**

To investigate if the completion of a recently developed treadmill-based wheelchair propulsion maximal progressive workload incremental test alters the integrity of the long head of the biceps and supraspinatus tendons using musculoskeletal ultrasound imaging biomarkers.

**Method:**

Fifteen manual wheelchair users completed the incremental test. Ultrasound images of the long head of the biceps and supraspinatus tendons were recorded before, immediately after, and 48 hours after the completion of the test using a standardized protocol. Geometric, composition, and texture-related ultrasound biomarkers characterized tendon integrity.

**Results:**

Participants propelled during 10.2 ± 2.9 minutes with the majority (*N* = 13/15) having reached at least the eighth stage of the test (speed = 0.8 m/s; slope = 3.6°). All ultrasound biomarkers characterizing tendon integrity, measured in the longitudinal and transversal planes for both tendons, were similar (*p* = 0.063 to 1.000) across measurement times.

**Conclusion:**

The performance of the motorized treadmill wheelchair propulsion test to assess aerobic fitness produced no changes to ultrasound biomarkers of the biceps or supraspinatus tendons. Hence, there was no ultrasound imaging evidence of a maladaptive response due to overstimulation in these tendons immediately after and 48 hours after the performance of the test.

## 1. Introduction

Individuals who exclusively use a wheelchair as their primary mode of locomotion experience prolonged nonactive sitting time [1, 2] with a reduction in, or cessation of, physical activity [3–5]. Over time, this day-to-day locomotion mode accelerates the development or exacerbation of chronic, complex, and interrelated secondary health problems, related to cardiorespiratory [6], musculoskeletal [7], and endocrine-metabolic health [8]. These chronic secondary health problems, often occurring simultaneously, are also frequently coupled with increased risk for nociceptive or neuropathic pain [9–11] or psychological morbidity [12, 13]. In turn, there is a reduction in functional skill and capacity, negative psychosocial side effects in long-term manual wheelchair users with a chronic spinal cord injury (MWU_SCI_) [14, 15] potentially increasing caregiver burden and the risk of premature mortality. Adapted physical capacity in MWU_SCI_ is a key factor in mitigating these life-altering health problems. Therefore, personalized and multifactorial interventions that provide MWU_SCI_ with the necessary resources, skills, and strategies for successful health behavior change remain an essential target for rehabilitation and physical activity professionals.

To develop personalized cardiorespiratory fitness training programs within this context and assess their impacts over time, cardiorespiratory fitness assessments among MWU_SCI_ are highly relevant [3, 16]. Due to limitations related to commonly used arm crank ergometer fitness test protocols, assessment protocols completed overground or on a motorized treadmill [17–19] have emerged as alternatives [20]. However, performing these task-specific testing protocols remains challenging, as manual wheelchair propulsion actively solicits upper limb muscles that are smaller, fatigue more easily and rapidly, and expend less energy, in comparison to larger lower limb muscles [21, 22]. In addition, when performing these task-specific testing protocols, upper limb muscles repeatedly generate elevated and rapidly rising forces applied to the hand rims until a general state of fatigue progressively develops. As an example, to estimate fitness of manual wheelchair users, a recently developed wheelchair propulsion treadmill-based test incrementally increases workload in small quantities by increasing the speed or slope of the treadmill [17, 18]. In fact, as the slope progresses from 0°, 2.7°, 3.6°, 4.8°, and to 7.1°, a biomechanical study has confirmed that the pushing frequency (duration of the pushing phase = −38%; duration of the recovery phase = −70%), the mean total (+201%) and tangential (+177%) forces applied to the hand rim, the net shoulder articular moments generated in flexion (+72%), adduction (+544%), and internal rotation (+255%), and the overall muscle utilization rates of the shoulder muscles (+238 to 400%) all increased progressively and significantly while mechanical efficiency fell by 12% [23, 24]. Altogether, these task-specific tests might represent an additional risk exposure for the development or exacerbation of upper limb secondary musculoskeletal impairments, especially at the shoulder where the soft tissue integrity is often altered [25]. Such an additional risk is disconcerting given the fact that upper limb injuries can affect up to 80% of this population (i.e., prevalence rate) and negatively impacts their daily activities [26]. Therefore, finding an equilibrium between the need to measure aerobic fitness and the risk of developing secondary musculoskeletal impairments remains fundamental among MWU_SCI_.

To determine if such an equilibrium exists, musculoskeletal ultrasound imaging and biomarkers of tendon integrity, which has progressed among MWU_SCI_ in the last decade [27, 28] may prove to be useful [29, 30]. This type of analysis contributed additional insights into the effects of fatiguing wheelchair propulsion, and wheelchair-related functional activity protocols, on the integrity of rotator cuff tendons. For instance, acute tendon reactivity [31] in response to an increased load has been characterized as an increase in vascularity [32] and a decrease in echogenicity [33].

The overall objective of this study is to investigate if the completion of a recently developed treadmill-based progressive workload incremental test [17, 18] alters the integrity of the biceps and supraspinatus tendons using ultrasound biomarkers of tendon integrity. To do so, musculoskeletal ultrasound images of the biceps and supraspinatus tendons will be recorded immediately before, immediately after, and 48 hours after the completion of a treadmill-based progressive workload incremental test to compare biomarkers of tendon integrity across these three times. The biceps tendon was selected since it is a biarticular muscle that contributes greatly (i.e., elevated relative muscular utilization ratio) to both net shoulder flexion and elbow flexion and extension moments during closed kinetic chain movements, such as the propulsive moment generated during the push phase of manual wheelchair propulsion [34], and frequently presents signs of tendinopathy among MWU_SCI_ [29]. As for the supraspinatus tendon, it was selected since it presents an elevated risk of mechanical impingement during propulsion [35] resulting in most part from the scapular kinematics [36], contributes greatly (i.e., elevated relative muscular utilization ratio) to the net shoulder moments during the push and recovery phases of manual wheelchair propulsion [34], and frequently present signs of tendinopathy among MWU_SCI_ [29]. Due to tendon adaptations from mechanical loading, it is anticipated that significant and clinically meaningful differences in tendon integrity will be found immediately after completion of the test, compared to immediately before. However, these same changes will not be found 48 hours after the completion of the test. This evidence will inform the risks of developing or exacerbating shoulder tendinopathy associated with the completion of a treadmill-based progressive workload incremental test.

## 2. Materials and Methods

### 2.1. Study Design

This hypothesis-driven secondary musculoskeletal ultrasound data collection builds on a one-group study design with repeated measurements recorded immediately before (*T*_1_) the treadmill-based progressive workload incremental test as well as immediately (*T*_2_) and 48 hours (*T*_3_) after its completion (i.e., satellite study). The primary data collection quantified the cardiorespiratory responses resulting from speed and slope increments during the test and proposed a predictive equation based on speed and slope for estimating peak oxygen uptake (VO_2peak_) in MWU_SCI_(i.e., parent study) [17, 18].

### 2.2. Participants

A consecutive convenience sample of 15 MWU_SCI_ was recruited. Participants were recruited via recruitment posters in key areas within the local rehabilitation hospital and associated outpatient clinics and announcements on social media platforms (i.e., Facebook). To be included in the study, potential participants were required to (1) use a manual wheelchair as their primary mean of mobility (≥4 hours per day), (2) report no history of cardiorespiratory disease, and (3) report no debilitating shoulder pain as measured with the Wheelchair User's Shoulder Pain Index (WUSPI) [37, 38], or any other secondary musculoskeletal impairments or complications affecting their trunk or upper extremities that could limit their ability to perform the experimental task. Potential participants were excluded if they presented any health condition(s) among the contraindications for cardiorespiratory fitness assessment and training according to American College of Sports Medicine (ACSM) standards [39] or responded positively to at least one item on the Physical Activity Readiness Questionnaire (PAR-Q) [40] without medical clearance for physical activity. Ethical approval was obtained from the Centre for Interdisciplinary Research in Rehabilitation of Greater Montreal (CRIR) Research Ethics Committees. Participants reviewed and signed informed consent forms before entering the study. For all participant-related information and collected data (including ultrasound images), all direct personal identifiers were replaced with a project-specific code to protect participant identity.

### 2.3. Experimental Task

The treadmill-based progressive workload incremental test was performed on a dual-belt motorized treadmill (Bertec Corporation, Columbus, Ohio, United States) adapted for safe manual wheelchair propulsion. Before the test, participants began with a five-minute warm-up on the treadmill to become familiarized with each speed and slope included in the test protocol. During the test, the exercise workload gradually intensified by increasing the treadmill slope (0°, 1.7°, 2.9°, 3.6°, 4.8°, 5.8° to 7.1°) or the speed (0.6 m/s, 0.8 m/s, and 1 m/s) every minute in a standardized manner (Table 1) [17, 18]. Participants were asked to propel their own MW until exhaustion. The test ended when the participants were unable to match the treadmill's speed during MW propulsion or if any signs or symptoms of exercise intolerance developed. Additional details about the test are available elsewhere.

### 2.4. Musculoskeletal Ultrasound

A single examiner (ML) conducted all musculoskeletal ultrasound assessments of the biceps and supraspinatus tendons of the nondominant shoulder using a Philips HD11XE machine and a 5 cm linear transducer (5-12 MHz) (Philips Medical Systems, Bothell, WA) according to standardized protocols (http://www.physiographie.umontreal.ca/protocoles/epaule) at *T*_1_, *T*_2_, and *T*_3_. The position of the probe was drawn directly on the skin of each participant with an indelible marker to assure its position remained identical across measurement across *T*_1_, *T*_2_, and *T*_3_. Three images of the biceps and supraspinatus tendons were recorded both in the longitudinal and transverse planes, respectively, to optimize reliability of and minimize the effects of measurement errors on all ultrasound biomarkers [27]. The settings of the machine were kept identical across participants and measurement times during the present study (e.g., depth = 4 cm; gain = 70 dB). All images of both tendons were recorded within a 10-to-15-minute period, with the order of tendons and image recording views randomized across participants. At *T*_2_, the elapsed time between the completion of the test and the time when image recording began (1-3 minutes) was the minimum time required to safely stop the treadmill and bring it back to 0° (i.e., no slope).

To record images of the biceps tendon, participants were seated upright in their personal wheelchair with their shoulder in a neutral position, elbow flexed to 90°, and forearm fully supinated while resting on a pillow. For longitudinal images, the transducer was positioned over the widest part of the tendon with the apex of the intertubercular groove of the humerus visible proximally on the ultrasound image (i.e., on the left side of the image by convention). For transverse images, the transducer was positioned so that the tendon and the bicipital groove, bounded medially and laterally by the lesser and greater tubercles, will be centered on the ultrasound image. To record images of the supraspinatus tendon, participants were seated in their MW with the palm of their hand first positioned on their lower back or wheelchair backrest (i.e., modified *Crass* position). For transverse images, the transducer was positioned to reveal the widest part of the supraspinatus tendon, with a view of the rotator interval, including a cross-sectional view of the biceps tendon. The ultrasound transducer was maintained perpendicular to the supraspinatus tendon fibers so that the tendon appeared hyperechoic and the adjacent humerus head cortex was brightly reflective to avoid tendon anisotropy. For longitudinal images, after participants moved their upper limb alongside their trunk into a neutral position, the ultrasound transducer was positioned perpendicular to the lateral aspect of the acromion in the frontal plane to visualize the supraspinatus tendon portion at the outlet of the subacromial space with the humeral head visible underneath.

### 2.5. Biomarkers of Tendon Integrity

**Figure 1:**
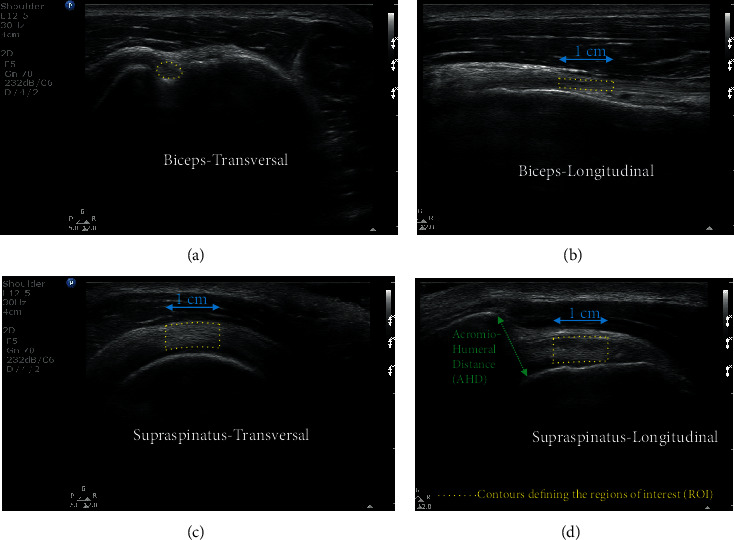
Illustraion of the regions of interests (ROIs) used to compute all biomarkers of tendon integrity.

All recorded images were analyzed using a custom interactive program developed using MATLAB image processing toolbox TM (The MathWorks Inc., Natick, MA). To extract all biomarkers of tendon integrity, a single evaluator (ML) manually outlined a 1 cm wide region of interest (ROI) (Figure 1) on the tendon being analyzed, using predefined anatomical landmarks.

Then, using the gray scale luminosity values (range = 0 to 255) of all pixels embedded within each ROI, geometric (i.e., thickness, cross sectional area), luminosity (i.e., echogenicity, variance, skewness, and entropy), and texture measures (i.e., contrast) were computed [27]. The mean distance between 100 equidistant points plotted on the upper and lower edges of the tendon were deemed the thickness of the ROI whereas the cross-sectional area represented the surface of a two-dimensional shape defined by the contour of the tendon. The echogenicity was computed as the mean of all pixel values within the ROI. Measures of variance (dispersion of pixel values around the mean), skewness (asymmetry of the median pixel value), entropy (randomness of pixel values), and contrast (quantity of local variation in grayscale between reference pixels and their neighbours) provided first- and second-order insights into the heterogeneity of the ROI. Finally, the acromiohumeral distance (AHD), considered a good indicator of the size of the subacromial space outlet, was computed as the shortest line (i.e., distance) possible between the acromion and the humeral head [41]. Additional information pertaining to these measures is available [27, 42].

### 2.6. Statistical Analyses

Descriptive statistics (i.e., mean and standard deviation (SD)) were used to characterize participant demographics. Since the sample size is limited and some biomarkers of tendon integrity were not normally distributed, nonparametric statistics (i.e., 25^th^, 50^th^ (median), and 75^th^ percentiles) were used to summarize the biomarkers of tendon integrity. To verify the hypothesis linked to the main objective, Friedman tests were conducted to compare results across *T*_1_, *T*_2_, and *T*_3_. Prior to doing so, participant's biomarker of tendon integrity measures that were ≥3 standard deviations away from the group mean were identified as an outlier and excluded from analyses. The alpha level was set at 0.05. All statistical analyses were performed with the SPSS statistics software, (version 26.0, IBM Corporation; Armonk, New York).

## 3. Results

### 3.1. Participants

A total of 15 MWU_SCI_ volunteered to participate in this study (Table 2). The median score on the WUSPI was 2/150 (min = 0; max = 26.4) while none of the participant exceeded a threshold of 3/10 for question 5 (“Pushing your chair for 10 minutes or more?”) and question 6 (“Pushing up ramps or inclines outdoors?”) on the WUSPI [37, 43] indicating none of the participants experienced debilitating shoulder pain.

### 3.2. Duration of the Progressive Workload Incremental Test

On average, participants completed 10.2 ± 2.9 stages of the incremental test, with the majority of participants (*N* = 13/15) having reached at least the eighth stage (speed = 0.8 m/s; slope = 3.6°).

### 3.3. Self-Reported Shoulder Pain

On average, participants self-reported a score of 0.5 ± 1.4 on the 10 cm visual analog scale for general shoulder pain. This score was identical before, after, and 48 h after the test, respectively.

### 3.4. Biomarkers of Tendon Integrity and Acromiohumeral Distance

All results are summarized in Table 3. Overall, biomarkers characterizing tendon integrity, measured in the longitudinal and transversal planes for the biceps and supraspinatus tendons, were not different (*p* = 0.063 to 1.000) across *T*_1_, *T*_2_, and *T*_3_. Likewise, AHD was also not different (*p* = 0.284) across the same measurement times.

## 4. Discussion

This study sets out to identify if changes in biceps and supraspinatus tendon integrity occur after a treadmill-based incremental workload test. The results of the present study only partially support the hypothesis initially formulated.

Contrary to our first hypothesis, no significant differences were found in biomarkers of tendon integrity recorded before and immediately after the completion of the maximal progressive workload incremental test. With additional mechanical stimulus and ensuing transient increase in microvascular volume, immediate and transient tendon adaptations [44] were anticipated. Moreover, the accumulation of large proteoglycans [45, 46], a major component of the extracellular matrix, causing an increase in water content and tendon dimension was also anticipated. These phenomena were expected to translate into a momentary hypoechogenicity [47, 48] and in turn changes to the composition-related biomarkers of tendon integrity, as the ability of the tendons to reflect ultrasound waves to form the desired image is being reduced. At the same time, potential delayed increases in collagen synthesis with mechanical loading, both in the tendon and in the space between the tendon and peritendinous sheath were also anticipated [45, 49, 50]. This increase in collagen synthesis could eventually translate into alterations of geometric- (e.g., increased thickness) and texture- (e.g., increased contrast) related biomarkers [27, 29, 51]. However, the mechanical constraints to which tendons are exposed during this test most likely reach intensities that maintain a dynamic state of equilibrium and potentially trigger favourable tendon adaptations. To this end, the performance of the test did not reveal any immediate maladaptative changes that could have resulted from an increase in inflammatory cytokines or markers of apoptosis [52, 53].

Previous ultrasound imaging studies examining changes in tendon integrity at the shoulder in response to wheelchair and wheelchair-related functional activities among long-term MWU_SCI_ have obtained conflicting results. For instance, Bossuyt et al. (2020) [54] investigated acute changes in the thickness, echogenicity, and contrast of the biceps and supraspinatus tendons following an intermittent intense wheelchair propulsion protocol (i.e., two times 40 seconds (s) treadmill propulsion at 1.11 meters at 25 Watt (W) and 45 W, three maximum push tests, and a maximum 15-meter overground sprint) among 50 MWU_SCI_. They found only an acute reduction in supraspinatus tendon thickness (–1.39 mm; 95% CI: -2.28; -0.51) immediately after the protocol. This finding highlights that the supraspinatus risk exposure for the development of secondary musculoskeletal impairments (including localized pain) is greater for the supraspinatus than the biceps. Further support for limited changes to tendon integrity comes from Van Drongelen et al. (2007) [51] who investigated acute changes in the thickness and echogenicity of the biceps and supraspinatus tendons following a wheelchair basketball or quad rugby game in MWU_SCI_. They revealed that there was a significant decrease in echogenicity in the biceps tendon only immediately after the game (mean duration 28.7 minutes). Interestingly, they suggested that exceeding a 30-minute playtime may be a key threshold to observe changes in tendon biomarkers. Such a threshold was not met in the present study and may explain in part the absence of change to biceps and supraspinatus tendons. Finally, Hogaboom et al. (2016) [55] investigated acute changes in the thickness, echogenicity, variance, and contrast of the biceps and supraspinatus tendons following the performance of a repeated sitting transfer protocol (i.e., three sets of six transfers with a 60-second break provided between each set) in MWU_SCI_. They also only revealed an acute increase in the biceps tendon thickness immediately after the protocol.

In contrast, two previous studies investigating a continuous propulsion task reported no effects on acute changes in biceps and supraspinatus tendon biomarkers or acromioclavicular and AHD in MWU_SCI_ [56, 57]. In fact, Gil-Agudo et al. (2016) [56] found no acute biceps and supraspinatus tendon change thickness changes and AHD during a submaximal continuous wheelchair propulsion protocol (i.e., treadmill wheelchair propulsion; mean duration = 14 min; mean speed = 0.3 m/s; mean power output = 51 W) whereas Collinger et al. (2010) [58] found no thickness, echogenicity, grayscale variance, entropy, contrast, energy, and homogeneity changes of the biceps and supraspinatus tendons following an intermittent maximal wheelchair propulsion protocol (i.e., 15-minute propulsion task composed of three 4-minute trials separated by 90 seconds of rest). Our results are in alignment with those two studies.

In line with our second hypothesis, 48 hours after the completion of the maximal progressive workload incremental test, no significant delayed differences in biomarkers of tendon integrity compared to immediately pre- or posttest was found. The typical physiological response to mechanical loading (i.e., transient increase in microvascular volume within the tendons and the accumulation of large proteoglycans) were expected to return to baseline values within 48 hrs postexercise. Moreover, the test was not expected to instigate significant structural change. In fact, changes that reduce tendon load capacity (maladaptive changes) were not expected since the load induce on the tendons during the test was personalized according to each participant's tolerance and they could also stop the test at any time, especially if shoulder pain developed (maladaptive changes). Moreover, changes improving tendon load capacity (i.e., beneficial adaptive changes) typically require regular loading over a prolonged period of time and was not investigated in the present study [58, 59]. Unfortunately, no previous studies focusing on the biceps and supraspinatus tendons in MWU_SCI_ has investigated whether the observed acute effects persisted or whether new effects emerged beyond the acute effects. Of note, changes in ultrasound-related biomarkers of tendon integrity of the lower limb have been documented after three to four days following the completion of maximal continuous running protocols, including treadmill-based protocols, in both humans and animals [60, 61].

Taken together, the absence of test-induced biomarker maladaptive changes after the completion of the wheelchair-based progressive workload incremental test supports its safety, especially with regards to increased risk of shoulder tendinopathy. Compared to other wheelchair-based protocols, the ability to warmup pretest, the relatively short test duration, and the graded effort likely modulate the risk to upper limb injury. These findings provide further support for the acceptability of this test in clinical practice and research protocols. Nonetheless, one needs to remain cautious as, when propelling, the shoulders are inevitably exposed to a progressive and quick elevation of rapidly raising mechanical loads during the test, especially as the slope and speed increments reach the maximal capacity of the MWU_SCI_ being tested [18]. Between-participant heterogeneity in tendon-based temporal response (i.e., reactivity) [29, 57] may also deserve future research attention as it may influence individual's response across different protocols. Likewise, delayed onset muscle soreness, which is typically evident at the musculotendinous junction before spreading throughout the muscle within 24-72 hours, deserves future investigation.

A few limitations that may influence the interpretation of the present results do require consideration. First, the small sample of participants and their heterogeneity (e.g., initial tendon characteristics) limit the potential to generalize the present results to the general population of manual wheelchair users. The small sample size also restricts the capability to complete subgroup analyses (e.g., sedentary versus physically active manual wheelchair users; low versus elevated relative shoulder muscular efforts) that are required to gain a better understanding of the tendon adaptation processes. Second, the variability during ultrasound image recording associated to the evaluator (e.g., location and orientation of the transducer, pressure applied on the transducer), the participants (e.g., upper extremity positioning, visibility of the landmarks outlined on the skin, level of physical activity prior to *T*_1_ or between *T*_2_ and *T*_3_), and the evaluator-participant interactions may have had an effect on the validity of the results. Third, the sensitivity to change threshold of the biomarkers of tendon integrity (including the resolution of the ultrasound imaging modality) and the 48 hrs time period elapsed between *T*_2_ and *T*_3_ may have been insufficient to confirm change in the biological integrity (i.e., composition and structure) of the tendon assessed. To this end, power doppler or elastography-related biomarkers could have provided additional insights regarding the vascularization and mechanical properties (e.g., stiffness) of the investigated tendons, respectively. Finally, these project-specific results solely reflect the biceps and supraspinatus tendon responses to a short duration and graded high intensity treadmill-based progressive workload incremental test (8-to-12-minute aerobic fitness test) and should not be generalized to other wheelchair-based testing or training protocols (e.g., high-intensity interval training) in MWU_SCI_. Investigating additional anatomical structures at the shoulder joints and potential complications (e.g., subacromial-subdeltoid bursa swelling, supraspinatus partial or complete tear, acromioclavicular joint inflammation) or other upper limb joints (e.g., wrist, elbow) is suggested to gain a better insight into the effects of the treadmill-based progressive workload incremental test.

## 5. Conclusions

The performance of the motorized treadmill propulsion test to assess maximal aerobic fitness produced no biceps and supraspinatus tendon changes observable with ultrasound biomarkers in MWU_SCI_. No evidence of maladaptive response due to overstimulation was observed in these tendons immediately after and 48 hours after the performance of the test. Further evidence is needed to fully understand the risks to the upper extremities associated with the performance of wheelchair-based maximal aerobic fitness tests or other training programs, especially high-intensity interval training program, among manual wheelchair users.

## Figures and Tables

**Table 1:** Treadmill-based incremental workload test for manual wheelchair users. The slope and speed of the treadmill were incrementally adjusted at each stage of the test until participants were unable to match the treadmill's speed.

Stages	1	2	3	4	5	6	7	8	9	10	11	12	13	14	15
Slope (°)	0°	0°	0°	1.7°	1.7°	2.9°	2.9°	3.6°	4.8°	4.8°	4.8°	5.8°	5.8°	7.1°	7.1°
Speed (m/s)	0.6	0.8	1	0.6	0.8	0.6	0.8	0.8	0.6	0.8	1	0.8	1	0.8	1

**Table 2:** Summary of participant characteristics.

Participant	Sex	Age (yr)	Weight (kg)	Type of SCI	AIS^∗^	Neurological level	Time since injury (years)	WUSPI Total score^∗∗^ (/150)	Last stage completed during test
1	M	38	56.8	Trauma	B	C7	7	0	8
2	M	33	67.6	Trauma	A	T12	16	2	16
3	M	63	96.7	Trauma	A	T10	10	13.2	8
4	M	39	58.6	Trauma	D	T6	17	0	13
5	M	19	na	Trauma	D	L1	1	0	8
6	M	26	70.4	Trauma	A	C7	2	0.3	8
7	M	21	72	Trauma	A	C6-C7	3	11.5	6
8	M	42	84.9	Trauma	C	T6	12	4	12
9	M	46	92.4	Trauma	A	T7	14	26.4	8
10	F	32	39.1	Nontrauma	A	T12	32	0.4	9
11	F	27	64.6	Trauma	A	T6	3	3.4	7
12	M	37	68.9	Trauma	A	T10	11	11	11
13	M	26	79.5	Trauma	A	T9	5	1.7	14
14	M	51	74.3	Trauma	A	T7	15	0	14
15	M	44	105.5	Trauma	A	T12	3	11.5	11
Mean		36.4	73.7		A	T6	13.5	5.7	10.2
SD		11.9	17.4				8.2	7.6	2.9

^∗^AIS: ASIA Impairment Scale; ASIA: American Spinal Cord Injury Association. A: no motor or sensory function is preserved below the neurological level; B: sensory function is preserved but no motor function below the neurological level; C: motor function is preserved below the neurological level, and more than half of the key muscles below the neurological level have a muscle grade < 3 out of 5 (manual muscle testing); D: motor function is preserved below the neurological level, and at least half of the key muscles below the neurological level have a muscle grade of ≥3 out of 5, E: motor and sensory function are normal. ^∗∗^WUSPI: Wheelchair User's Pain Index.

**Table 3:** Summary of biomarkers of tendon integrity for the long head of the biceps and supraspinatus tendons and of the acromiohumeral distance measured before (pre), immediately after (post), and 48 hrs after the wheelchair test.

			Pre			Post			48 hrs				
			Percentiles			Percentiles			Percentiles			Comparisons	
Structure	Measure		25th	50th (median)	75th	25th	50th (median)	75th	25th	50th (median)	75th	*p* value	Khi-Carré
Biceps	Transverse	Thickness	2.817	3.358	4.027	3.06	3.478	4.006	3.337	3.575	4.462	0.247	2.8
		Area	20.369	26.384	32.077	21.177	26.764	34.507	25.893	30.952	32.039	0.063	5.538
		Echogenicity	59.904	62.844	104.846	57.922	71.742	96.956	50.783	68.179	78.333	0.936	0.133
		Skewness	0.354	0.518	0.627	0.16	0.386	0.493	0.263	0.535	0.608	0.819	0.4
		Variance	237.82	332.285	560.691	309.479	412.658	512.996	291.032	355.446	460.241	0.807	0.429
		Contrast	0.48	0.7	1.412	0.542	0.809	1.587	0.457	0.645	0.991	0.751	0.571
		Entropy	5.806	5.988	6.324	6.007	6.154	6.271	5.897	6.042	6.167	0.936	0.133
	Longitudinal	Thickness	3.312	3.724	4.255	3.264	3.979	4.495	3.445	4.019	4.796	0.189	3.333
		Echogenicity	64.663	83.291	92.942	71.665	89.9	93.86	58.273	63.045	88.264	0.247	2.8
		Skewness	0.385	0.497	0.705	0.348	0.409	0.762	0.4	0.503	0.826	1	0
		Variance	226.077	298.044	374.762	229.233	317.033	436.665	182.577	280.012	404.772	0.807	0.429
		Contrast	0.55	0.897	1.247	0.557	1.081	1.422	0.461	0.675	1.117	0.607	1
		Entropy	5.828	5.921	6.128	5.725	5.989	6.177	5.603	5.96	6.229	0.627	0.933
Supraspinatus	Transverse	Thickness	5.399	6.699	7.319	5.75	6.561	7.468	5.689	6.546	7.128	0.247	2.8
		Echogenicity	63.892	72.676	78.858	56.816	64.838	71.624	59.606	67.731	71.066	0.344	2.133
		Skewness	0.308	0.748	0.875	0.4	0.489	0.841	0.301	0.632	0.694	0.627	0.933
		Variance	524.367	570.404	873.071	435.754	551.808	704.346	424.84	576.67	749.165	0.247	2.8
		Contrast	0.532	0.673	0.863	0.524	0.613	0.696	0.564	0.612	0.678	0.395	1.857
		Entropy	6.277	6.383	6.6	6.199	6.31	6.42	6.191	6.4	6.478	0.42	1.733
	Longitudinal	Thickness	3.17	4.54	7.063	3.047	4.216	6.537	3.339	4.772	6.505	0.085	4.933
		Echogenicity	41.174	49.502	55.095	41.252	50.625	68.397	46.578	56.56	69.437	0.247	2.8
		Skewness	0.553	0.755	0.861	0.439	0.626	0.816	0.412	0.575	0.724	0.168	3.571
		Variance	358.921	568.832	695.991	371.866	515.437	732.862	504.097	677.26	911.744	0.223	3
		Contrast	0.301	0.457	0.656	0.361	0.5	0.696	0.388	0.552	0.743	0.344	2.133
		Entropy	6.048	6.366	6.493	6.101	6.335	6.589	6.225	6.507	6.657	0.344	2.133
Acromiohumeral	Distance	Transverse	1.013	1.073	1.1	1.01	1.103	1.167	0.91	1.05	1.173	0.284	2.517

## Data Availability

The datasets generated and/or analyzed during the current study are not publicly available due to data safety considerations but remains available from the corresponding author on reasonable request upon approval of the Research Ethics Committee of the Centre for Interdisciplinary Research in Rehabilitation of Greater Montreal.

## Abbreviations

ACSM: American College of Sports Medicine
AHD: Acromiohumeral distance
BL: Biceps longitudinal
BT: Biceps transverse
CRIR: Centre for Interdisciplinary Research in Rehabilitation of Greater Montreal
CSA: Cross-sectional area
LHB: Long head of the biceps
MW: Manual wheelchair
MWU_SCI_: Manual wheelchair user with spinal cord injury
PAR-Q: The Physical Activity Readiness Questionnaire for everyone
QUI: Quantitative ultrasound imaging
QUS: Quantitative ultrasound
RC: Rotator cuff
ROI: Region of interest
SCI: Spinal cord injury
SD: Standard deviation
SEM: Standard error of measurement
SS: Supraspinatus
SST: Supraspinatus transverse
SSL: Supraspinatus longitudinal
UI: Ultrasound image
WUSPI: Wheelchair User's Shoulder Pain Index.
